# An emerging picture of the seed desiccome: confirmed regulators and newcomers identified using transcriptome comparison

**DOI:** 10.3389/fpls.2013.00497

**Published:** 2013-12-11

**Authors:** Emmanuel Terrasson, Julia Buitink, Karima Righetti, Benoit Ly Vu, Sandra Pelletier, Julia Zinsmeister, David Lalanne, Olivier Leprince

**Affiliations:** ^1^Université d'Angers, UMR 1345 Institut de Recherche en Horticulture et Semences, SFR 4207 QUASAVAngers, France; ^2^Institut National de la Recherche Agronomique, UMR 1345 Institut de Recherche en Horticulture et Semences, SFR 4207 QUASAVAngers, France; ^3^Agrocampus Ouest, UMR 1345 Institut de Recherche en Horticulture et Semences, SFR 4207 QUASAVAngers, France

**Keywords:** ABI3, ABI5, ABA, desiccation tolerance, transcription factor, seed, transcriptome, *Medicago truncatula*

## Abstract

Desiccation tolerance (DT) is the capacity to withstand total loss of cellular water. It is acquired during seed filling and lost just after germination. However, in many species, a germinated seed can regain DT under adverse conditions such as osmotic stress. The genes, proteins and metabolites that are required to establish this DT is referred to as the desiccome. It includes both a range of protective mechanisms and underlying regulatory pathways that remain poorly understood. As a first step toward the identification of the seed desiccome of *Medicago truncatula*, using updated microarrays we characterized the overlapping transcriptomes associated with acquisition of DT in developing seeds and the re-establishment of DT in germinated seeds using a polyethylene glycol treatment (−1.7 MPa). The resulting list contained 740 and 2829 transcripts whose levels, respectively, increased and decreased with DT. Fourty-eight transcription factors (TF) were identified including *MtABI3*, *MtABI5* and many genes regulating flowering transition and cell identity. A promoter enrichment analysis revealed a strong over-representation of ABRE elements together with light-responsive *cis*-acting elements. In *Mtabi5 Tnt1* insertion mutants, DT could no longer be re-established by an osmotic stress. Transcriptome analysis on *Mtabi5* radicles during osmotic stress revealed that 13 and 15% of the up-regulated and down-regulated genes, respectively, are mis-regulated in the mutants and might be putative downstream targets of *MtABI5* implicated in the re-establishment of DT. Likewise, transcriptome comparisons of the desiccation sensitive *Mtabi3* mutants and hairy roots ectopically expressing *MtABI3* revealed that 35 and 23% of the up-regulated and down-regulated genes are acting downstream of *MtABI3*. Our data suggest that ABI3 and ABI5 have complementary roles in DT. Whether DT evolved by co-opting existing pathways regulating flowering and cellular phase transition and cell identity is discussed.

## Introduction

Understanding osmotic stress responses is one of the most important topics in plant science as this stress causes adverse effects on crop yield and quality (Hirayama and Shinozaki, 2010). For example, the recent drought waves that occurred in Europe in 2003 and in most of the USA in 2012 caused an estimated 30% reduction in primary productivity (Ciais et al., 2005; Claeys and Inzé, 2013). With the prospect of climate changes that are expected to worsen water limitations in the future, these figures suggest that major progress in understanding how plants cope with drought stress remains to be made. Given these agricultural challenges, it is remarkable that there exist biological mechanisms that allow complete desiccation with loss of viability (Hoekstra et al., 2001; Moore et al., 2008; Leprince and Buitink, 2010; Gechev et al., 2012; Gaff and Oliver, 2013). Desiccation tolerant seeds and leaves have acquired mechanisms that enable them to safely experience decreasing water potentials at which drought tolerant tissues are barely surviving (i.e., > −3 MPa for cell protoplasts). Therefore, it is argued that understanding the molecular processes leading to desiccation tolerance (DT) in seeds and resurrection plants might help the design and production of drought tolerant crops (Bartels and Sunkar, 2005; Moore et al., 2009; Rodriguez et al., 2010; Oliver et al., 2011; Gechev et al., 2012).

The desiccome can be referred to as the set of genes, proteins and metabolites that are required for DT (Leprince and Buitink, 2010). They are part of the protective mechanisms that can be grouped in at least three types that act synergistically (for reviews see Hoekstra et al., 2001; Tunnacliffe and Wise, 2007; Moore et al., 2008, 2009; Mönke et al., 2012; Leprince and Buitink, 2010; Gaff and Oliver, 2013): (1) protection by stabilization of membranes and proteins by non-reducing sugars, late embryogenesis abundant (LEA) proteins and heat shock protein (HSP), (2) protection against oxidative damage by a range of antioxidant compounds such as tocopherols, glutathione, together with a coordinated response of metabolism during drying and (3) protection against structural stresses imposed by drying such as cell wall modification, reorganization of endomembranes and cytoskeleton, vacuolization and chromatin condensation. The desiccome also includes the regulatory mechanisms and signaling pathways controlling the induction of these protective mechanisms leading to DT. However, these pathways are still poorly understood.

*Abscisic acid* (*ABA*) has been long known to play a prominent regulatory role in DT. In several species of resurrection plants, exogenous ABA induces DT [reviewed by Gaff and Oliver (2013)] whereas in seeds, genetic screens for ABA insensitivity during germination has led to the discovery of *abscisic acid insensitive 3* (*abi3*) mutants. Severe alleles of *abi3* mutants of several species, including *Arabidopsis* (Ooms et al., 1993) and *Medicago truncatula* (Delahaie et al., 2013) produce desiccation-sensitive seeds and have reduced expression of many genes related to DT like LEA genes. An ABI3 ortholog has been found to activate LEA genes in the moss *Physcomitrella patens* (Yotsui et al., 2013), suggesting that ABI3 is part of an evolutionarily conserved regulatory network. *ABI3* encodes a transcription factor belonging to the B3 domain-containing family and its function in the ABA signaling pathway in seeds is well characterized (Cutler et al., 2010; Hauser et al., 2011; Nakashima and Yamaguchi-Shinozaki, 2013). Transcriptional targets of ABI3 have been identified based either on transcript analysis in loss-of-function mutants or over-expressing transgenics (Nakashima et al., 2006) or through genome wide chromatin immunoprecipitation (Mönke et al., 2012). They include oleosins, LEA proteins and storage proteins. However, the experimental set up of these studies did not consider a putative link with DT.

To date, the ABA-signaling pathway leading to DT is mainly inferred from the knowledge gained from genetic and biochemical studies in drought tolerance in vegetative tissues and seed development. Indeed, the backbone of the ABA core signaling pathway, including positive and negative regulators involved in the response to osmotic stress in vegetative tissues, is very similar to that involved in seed maturation during which DT is acquired (Finkelstein et al., 2005; Fujita et al., 2011; Hauser et al., 2011; Nakashima and Yamaguchi-Shinozaki, 2013). In seeds and seedlings, basic leucine zipper (bZIP) transcription factors (TF) belonging to the ABA Responsive Element Binding Factors (AREB/ABF) and ABA INSENSITIVE 5 (ABI5) clade represent key TF controlling ABA-responsive gene expression such as LEA genes by interacting with ABA responsive regulatory elements (ABRE) in their promoter region. In *Arabidopsis*, promoters of genes with transcript levels that accumulate during maturation drying and that are stored in the dry seeds exhibit an enrichment in ABRE elements (Nakabayashi et al., 2005). ABI5 is considered as a crucial player in ABA signaling (Fujita et al., 2011). ABI5 appears to be courted by multiple proteins, thereby forming a complex interactome (Lindemose et al., 2013). There is also a large body of evidence suggesting that ABI5 forms a complex with ABI3 to regulate the expression of downstream genes with ABI3 acting as an accessory enhancer of transcription (Nakamura et al., 2001; Lindemose et al., 2013). It is suggested that such interaction occurs only during seed maturation (Finkelstein et al., 2005). ABI5 exerts a positive regulation of *EM1* and *EM6* genes (Nakamura et al., 2001; Finkelstein et al., 2005) both encoding LEA proteins whose abundance correlates with DT in developing and germinating seeds of *Medicago* (Boudet et al., 2006; Chatelain et al., 2012). However, null alleles of ABI5 apparently produce desiccation-tolerant seeds in *Arabidopsis*. During seedling establishment, ABI5 controls an ABA- or osmotic stress induced post-germinative growth arrest that is accompanied by an increased drought tolerance (Lopez-Molina et al., 2001, 2002). Therefore, the precise role of ABI5 in DT, if any, remains to be elucidated.

In this study, we investigated the regulatory components involved in DT in *M. truncatula* seeds by revisiting the transcriptome changes associated with the acquisition of DT during seed maturation and the re-establishment of DT in emerged radicles upon an osmotic treatment using a PEG solution at −1.7 MPa (Buitink et al., 2006; Verdier et al., 2013). We used an updated Nimblegen slide containing the almost complete *M. truncatula* genome. One of the identified TF that was further characterized for its role in DT was *MtABI5*. New putative ABI5-regulated target genes that are involved in DT were identified from a transcriptome analysis. In addition, to identify ABI3-related genes involved in DT, we also took advantage of recently obtained transcriptome data on *Medicago Mtabi3* seeds and hairy roots ectopically expressing *MtABI3*. The comparison of these five transcriptomes provides insights into the desiccome that is regulated by ABI3 and ABI5 and identifies new regulators that might play a role in DT in seeds.

## Materials and methods

### Plant materials and physiology

Plants of *Medicago truncatula* ssp. *tricycla* (R108) were grown in a sterile mix of vermiculite and soil in a growth chamber at 24°C/21°C, 16 h photoperiod at 200 μM m^−2^ s^−2^. Seeds were harvested upon pod abscission and stored at 5°C at 43% relative humidity (RH) until use. Two *M. truncatula* mutants with *Tnt1* insertions in the *MtABI5* gene (NF4383, hereafter referred to as *Mtabi5-1* and NF3376, *Mtabi5-2*) were obtained from the Samuel Noble Foundation (Oklahoma, USA). *Tnt1* insertions in both mutants were verified by PCR according to Delahaie et al. (2013) using forward and reverse primers ATGGTGGTAAGAGAAGGTGAGAT and AGCAGCAAGATCTAGAGCCAGA, respectively. Mutant and wild-type lines (R108) were multiplied in a growth chamber according to Chatelain et al. (2012). The Mt*abi5-1* line was backcrossed twice.

To determine ABA sensitivity, batches of 30–50 seeds were scarified with sand paper and imbibed on filter paper on a range of ABA concentrations (mixed isomers, Sigma, St Louis, MO, USA) at 20°C in the dark. ABA was dissolved in methanol prior to dilution in water. Control seeds were imbibed in the MeOH concentration corresponding to the highest ABA concentration (0.5% MeOH). Germination was scored after 14 days.

The re-establishment of DT after germination was performed according to Buitink et al. (2003). Germinated seeds with a protruded radicle length of 2.7–2.9 mm (thereafter referred to as 2.7 mm) were selected and submitted to an osmotic treatment by incubation in a PEG 8000 solution (−1.7 MPa) at 10°C in the dark. After 72 h, seeds were removed from the PEG solution and rinsed thoroughly. Three replicates of 50 radicles before and after PEG treatment from WT and *Mtabi5* mutants were excised and frozen for RNA extraction. DT of germinated, untreated and PEG-treated germinated seeds was assessed by drying 50 seeds for each condition for 3 days at 20°C under an air flow at 42% RH. Radicles were considered desiccation-tolerant when they resumed growth upon re-imbibition.

### Bioinformatic analyses

A blast was performed on the *Medicago* IMGAG Mt3.5.1 version using the *Arabidopsis* ABI5 amino acid sequence (At2g36270.1) to retrieve homologous genes (score *e* < 1E-20). An unrooted tree was constructed with the nine identified *MtABI5*-like sequences and the *Arabidopsis* ABI5 using the sequence alignment program ClustalW (http://www.ebi.ac.uk/Tools/msa/clustalo/) and presented using Treeview (Page, 1996). Gene lists were analyzed for enrichment in gene ontology (GO) terms using the Singular Enrichment Analysis (SEA) tool of AgriGO (http://bioinfo.cau.edu.cn/agriGO/) with a Chi-square statistical test method and the Yekutieli multi-test adjustment method (Du et al., 2010). For the large dataset of down-regulated transcripts related to DT, Plant Slim GO analysis was used. Data lists were analyzed using the *Arabidopsis* TAIR9 background based on homology with the *Medicago* sequences since the *M. truncatula* genome background in AgriGO is incomplete.

For *cis*-element enrichment analysis of the DT-UP list, 1.5 Kb promoter regions upstream of the translational start of the 382 IMGAG Mt3.5.1 sequences were retrieved using the Legoo gateway (https://www.legoo.org/). Promoter sequences were analyzed using the PLACE database resource, (http://www.dna.affrc.go.jp/PLACE/signalup.html). The frequency of a specific motif in the promoters of genes was tested against a background frequency generated using a randomized sample of 1470 *Medicago genes*. The significance of the enriched *cis*-elements was calculated using a Chi-square test against the background sample.

### RNA extraction and microarray analysis

Total RNA were extracted using the nucleospin RNAplant kit (Macherey Nagel, Düren, Germany) and 400 ng were amplified using the Ambion messageAmp II (Ambion, Austin TX) following manufacturer's instructions. Five μg of amplified RNA were retro-transcribed with 400 U of Superscript II reverse-transcriptase (Invitrogen Corp., Carlsbad, CA) and labeled with 1.5 mmol of Cyanine-3 (Cy3) or Cyanine-5 (Cy5) (Interchim, France) then purified using NucleoSpin Gel and PCR Clean-up column kits (Macherey-Nagel, GmbH and Co. KG, Germany). Purified and labeled cDNA were quantified using a NanoDrop ND-1000. Corresponding Cy3- and Cy5 labeled samples (30 pmol) were combined and co-hybridized to Medtr_v1.0 12x135K arrays according to Verdier et al. (2013). The Medtr_v1.0 chip was *in situ* synthesized by Nimblegen (Madison, WI) and contains 102,123 60-mer oligoprobes that were designed from an intermediate annotation of the *M. truncatula* genome, containing one probe per sequence of the IMGAG Mt3.5.1 version and additional sequences from RNAseq (Verdier et al., 2013). Three biological replicates were analyzed per comparison using the dye-switch method, and statistical analysis on the gene expression data was performed according to Verdier et al. (2013). Probes with a *P* < 0.01 and a log ratio>|1| were considered differentially expressed.

### Data submission

Nimblegen microarray data were deposited on the NCBI GEO database [(Barrett et al., 2012); accession GSE51830]. Data on the ectopic expression of *MtABI3* in hairy roots discussed in this publication have been deposited in NCBI Gene Expression Omnibus (Edgar et al., 2002) and are accessible through GEO Series accession number GSE44291 (http://www.ncbi.nlm.nih.gov/geo/query/acc.cgi?acc=GSE44291).

### Quantitative PCR

For real time RT-PCR, 1 μg total RNA was reverse-transcribed using Quantitect Reverse Transcription kit (Qiagen, Courtaboeuf, France) and qPCR was performed on a CFX96 Real-Time Detection System (Bio-Rad Laboratories, Hercules, CA) using the manufacturer's instructions with SsoFast Eva Green Supermix (Bio-Rad Laboratories, Hercules, CA). For Medtr5g010210.1 (GCL210, glutamate cysteine ligase), 5′TCTACGGCCAAAACTGTTGC3′ and 5′CGTAACGTACCTTGGGCACTA3′ were used as forward and reverse primers, respectively. For Medtr5g010230.1 (GCL230), 5′TCCTGGATATGATTGCTGATTGGA3′ and 5′AGAGTCTTTAAACCGGTGAAAGGA3′ were used as forward and reverse primers, respectively. The reference gene used for normalization was *MSC27* (Bolingue et al., 2010). Relative expression levels were calculated using the comparative 2∆(Ct) method. Each data point represents the mean of three biological replicates.

## Results and discussion

### Identification of the transcriptional desiccome using two physiological models of desiccation tolerance

**Figure 1:**
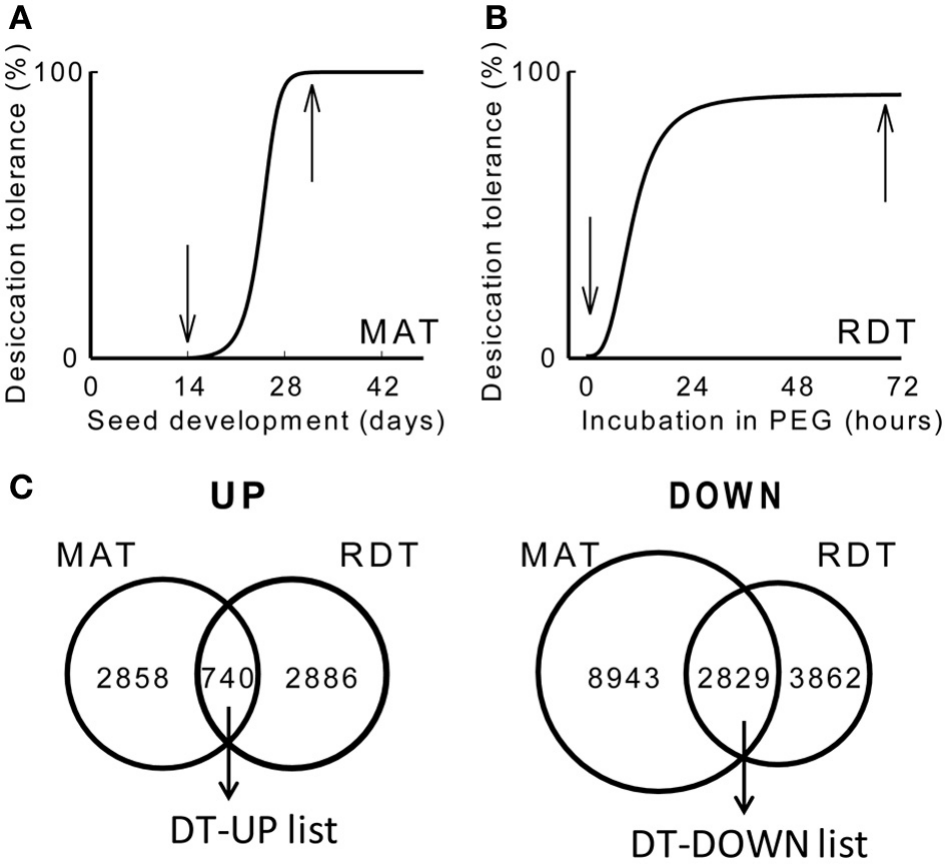
**Changes in desiccation tolerance (DT) and the associated desiccome in *Medicago truncatula*.** (**A,B**) Schematic representation of the acquisition of DT during seed maturation (MAT) at the indicated days after pollination (DAP) [**A**, curve taken from Chatelain et al. (2012)] and the re-establishment of DT (RDT) in emerged radicles of germinated seeds during the incubation in a polyethylene glycol solution (PEG) at −1.7 MPa [**B**, curve taken from Buitink et al. (2006)]. Arrows indicate the time at which samples were taken for the transcriptome analysis. **(C)** Venn diagrams comparing the overlapping transcripts whose levels are increased (UP) or decreased (DOWN) during the transition from a desiccation-sensitive to -tolerant stage during maturation (MAT) and PEG incubation (RDT). Genes were considered statistically different when −1 < M(log2) > 1 and *P* < 0.01.

To obtain an overview of the genes implicated in DT, we produced two transcriptome data sets using an updated *Medicago* Nimblegen microarray: one on the acquisition of DT during maturation (GEO database accession GSE49350), and a second one on the re-establishment of DT during PEG incubation. To identify genes that are differentially expressed during the acquisition of DT, transcript levels were compared between 14 days after pollination (DAP), when seeds are still desiccation sensitive, and 32 DAP, when 100% of the seeds have acquired their DT (Figure 1A). Data analysis identified 3626 and 14601 transcripts that increased or decreased at least two-fold, respectively, between 14 and 32 DAP. Next, we characterized the transcriptome in relation to the re-establishment of DT. DT is lost following germination after the radicle is protruded, first in the radicles and then in the cotyledons. However, it is possible to re-establish DT in these protruded radicles by an osmotic stress treatment using a PEG solution at −1.7 MPa (Buitink et al., 2003; Faria et al., 2005). We performed a transcriptome analysis on 2.7 mm long protruded radicles before and after 72 h of PEG incubation, corresponding to desiccation sensitive and -tolerant tissues, respectively (Figure 1B). A total of 3598 and 6691 transcripts increased or decreased significantly (ratio log2 > 1 or <-1, *P* < 0.01). Both datasets were compared to each other to identify overlapping transcripts (Figure 1C). This resulted in the identification of 740 transcripts that positively correlate with DT (hereafter referred to as the DT-UP list) and 2829 transcripts that are down-regulated when DT is installed (DT-DOWN list) (Figure 1C) (Supplementary Table S1). It should be noted that some redundancy exists between the probes. Some of them are recognized by the same gene transcript, which can only be removed when the assembly of the *M. truncatula* genome is completed.

The DT associated transcriptome of *Medicago* corresponding to a more complete dataset using updated slides, was compared to the recently obtained data set on a similar system of re-establishment of DT in *Arabidopsis* (Maia et al., 2011). Compared to this study, the number of transcripts overlapping between *Arabidopsis* and *Medicago* increased from 22 (48 transcripts) to 77% (115) of the total amount of up-regulated genes in *Arabidopsis* during re-establishment of DT and from 13 (49 transcripts) to 50% (139) for the down-regulated genes. These values reinforce the idea that core mechanisms and key regulators involved in DT are conserved across species, despite the fact that whole seeds were used for *Arabidopsis* and only radicles for *M. truncatula* (Maia et al., 2011).

**Figure 2:**
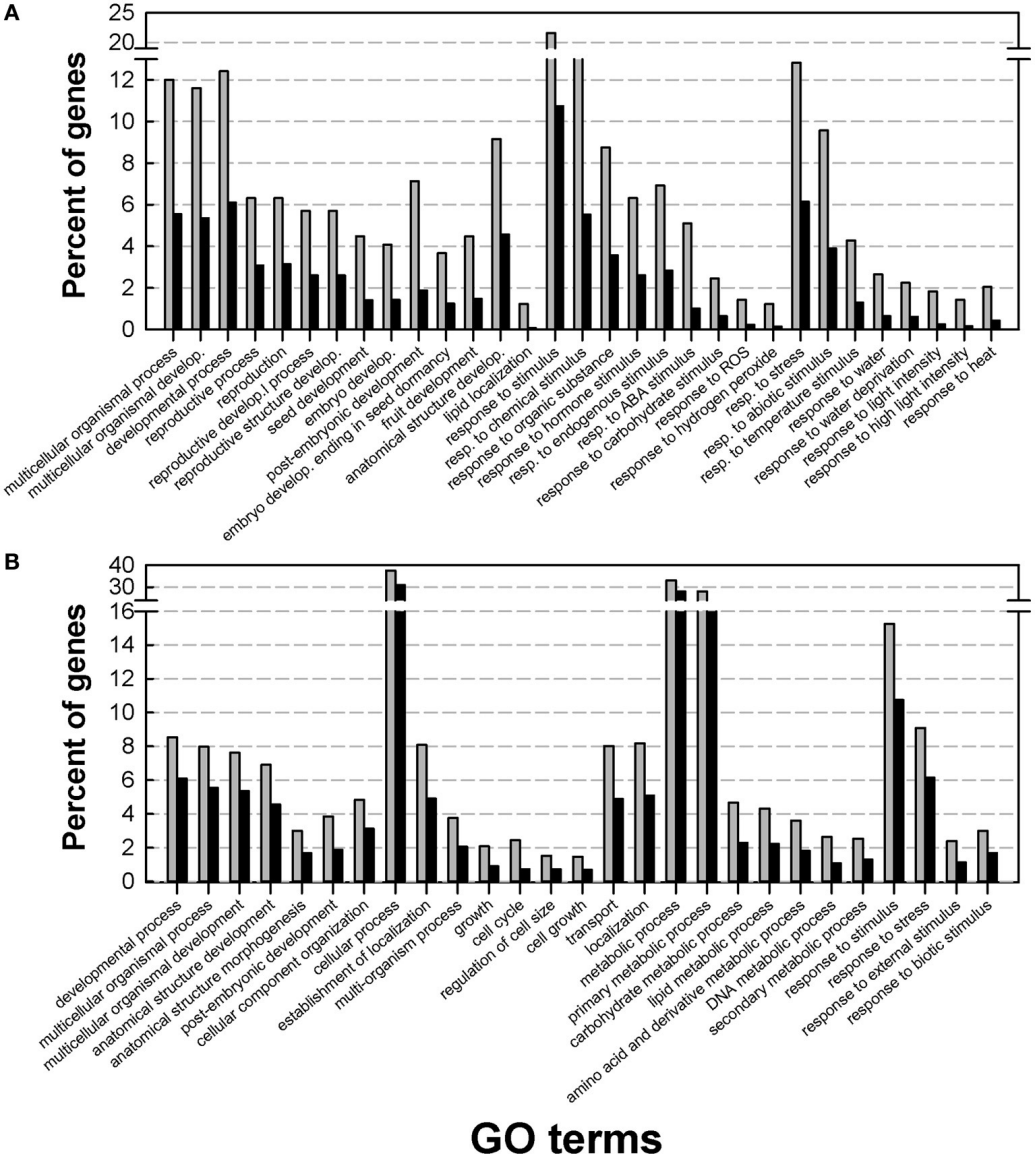
**Gene Ontology (GO) enrichment analysis of the desiccation tolerant UP list (A) and desiccation tolerant DOWN list (B).** Analysis was performed on the DT-UP and DT-DOWN list from Figure 1 using AgriGO with the *Arabidopsis* TAIR9 background applying a Chi^2^ statistical test with the Yekutieli multi-test adjustment method. Gray bars: input genes, black bars background genes.

To gain insight into the *Medicago* DT-related transcriptome, a GO enrichment analysis was performed using agriGO. Within the up-regulated genes, the main enriched GO terms belonged to three broad categories: developmental processes, endogenous stimuli and external stimuli and stress (Figure 2A). A closer look at these GO terms identified categories previously described by Maia et al. (2011) in the relation to the re-establishment of DT in germinated seeds, such as post-embryonic development, response to ABA, and response to water deprivation. The latter two GO terms were also enriched in the transcriptome of dehydrating leaves of the resurrection plant *Craterostigma plantagineum* (Rodriguez et al., 2010), reinforcing the importance of ABA as a core regulator of DT. Our data also revealed an important and previously un-described category in the DT-UP list, namely “anatomical structure development.” Apart from genes that have been discovered previously in relation to DT, such as LEA genes, oleosins, or 1-cys peroxyredoxin, this GO category revealed genes whose *Arabidopsis* homologs are involved in the regulation of cell growth, cell formation or identity. This category also contains several probes whose homologs in *Arabidopsis* impose maternal effects on seed development such as DEMETER (responsible for endosperm maternal-allele-specific hypomethylation at the MEDEA gene) and MATERNAL EFFECT EMBRYO ARREST 11 and 14 (MEE11, MEE14), whose mutants are embryo lethal because of endosperm development arrest (Pagnussat et al., 2005). It is long recognized that many seed traits such as weight, germination and even longevity (i.e., survival in the dry state for a period of time) are under the influence of parent-of-origin effects (Blödner et al., 2007; Donohue, 2009; Kochanek et al., 2011). Since the acquisition of DT is essential to complete the plant life cycle, these data raise the intriguing question whether maternal effects might also influence the transcriptional part of the desiccome.

The GO categories of the DT-down list were related to cell cycle, growth and regulation of cell size (Figure 2B). For instance, they contained genes associated with the modification of cell walls (cellulose synthase, expansins, ATGH9B1 (*ARABIDOPSIS THALIANA* GLYCOSYL HYDROLASE 9B1) and the cytoskeleton. Also GO categories related to transport and metabolism were found to be enriched in the down-regulated desiccome.

### Characterization of transcriptional regulators and regulatory *cis*-elements in the up-regulated desiccation-tolerance related transcriptome

To gain further insight into the transcriptional machinery regulating DT independently of the developmental context, 48 TF that were manually curated using Ensembl and Plantdtb (http://planttfdb.cbi.pku.edu.cn) were retrieved from the up-regulated gene list (Table 1). AP2/EREBP, bHLH, and bZIP family members were found to be the most abundant TF. Twenty-two percent of these TF correspond to *Arabidopsis* homologs associated with freezing, cold and drought response and/or mediating an ABA response such as members of the DREB (DRE binding protein) subfamilies (Table 1, bold characters) as previously described (Fujita et al., 2011).

**Table 1:** **Transcription factors identified in the desiccome of *Medicago truncatula* (UP-DT list, see Figure 1) and homologs in *Arabidopsis thaliana***.

	***Medicago truncatula***	***Arabidopsis thaliana***			**Classification**	**Putative regulator**
**Sequence ID**	**Protein name**	**AGI**	**Protein name**	***e*-value**		
**MEDTR1G019110.1**	**Ethylene-responsive transcription factor ERF008)**	**AT2G23340.1**	**DEAR3 (DREBA5)**	**9.00E-42**	**AP2/EREBP**	**ABI3**
**MEDTR1G023170.1**	**Dehydration-responsive element-binding protein 2D (ERF071)**	**AT1G75490.1**	**DREB subfamily A2**	**1.00E-41**	**AP2/EREBP**	**ABI3**
MEDTR1G079630.1	Dehydration-responsive element-binding protein 2F	AT3G57600.1	DREB subfamily A-2	1.00E-17	AP2/EREBP	ABI3
**MEDTR4G127930.1**	**AP2-like ethylene-responsive transcription factor AIL5**	**AT5G57390.1**	**CHO1**	**1.00E-125**	**AP2/EREBP**	**ABI3**
**MEDTR7G070220.1**	**Dehydration-responsive element-binding protein 2C**	**AT2G40340.1**	**DREB2C,AtERF48**	**7.00E-39**	**AP2/EREBP**	**ABI3**
*MEDTR2G098180.1*	*AP2-like ethylene-responsive transcription factor PLT2*	*AT1G51190.1*	*PLT2*	*1.00E-162*	*AP2/EREBP*	*NI*
**CU571152_1013.1**	**Ethylene-responsive transcription factor ERF109**	**AT4G34410.1**	**RRTF1**	**2.00E-26**	**AP2/EREBP**	**ABI3**
MEDTR3G062440.1	Pathogenesis-related genes transcriptional activator PTI6	AT4G27950.1	CRF4	1.00E-22	AP2/EREBP	*NI*
MEDTR5G016750.1	Ethylene-responsive transcription factor ERF010	AT5G67190.1	DEAR2 (DREBA5)	2.00E-46	AP2/EREBP	*NI*
*MEDTR4G060950.1*	*LOB domain-containing protein 11*	*AT2G28500.1*	*LBD11*	*8.00E-60*	*AS2*	*NI*
**MT3.5.1S031063**	**ABI3**	**AT3G24650.1**	**ABI3,SIS10**	**1.00E-134**	**B3**	***NI***
*MT3.5.1S022226*	*Transcription factor bHLH80*	*AT1G35460.1*	*FBH1*	*6.00E-41*	*bHLH*	*ABI3/ABI5*
MT3.5.1S041843	Transcription factor bHLH135	AT3G28857.1	PRE5	9.00E-33	bHLH	ABI5
*MT3.5.1S054079*	*Transcription factor bHLH130*	*AT2G42280.1*	*FBH4*	*7.00E-78*	*bHLH*	*ABI5*
*MEDTR1G105280.1*	*Transcription factor bHLH130*	*AT2G42280.1*	*FBH4*	*6.00E-53*	*bHLH*	*NI*
*MEDTR2G104550.1*	*Transcription factor bHLH25*	*AT4G37850.1*	*BHLH25*	*3.00E-38*	*bHLH*	*NI*
*MEDTR4G097950.1*	*Transcription factor bHLH25*	*AT4G37850.1*	*BHLH25*	*7.00E-43*	*bHLH*	*NI*
*MEDTR3G101810.1*	*Transcription factor HEC2*	*AT3G50330.1*	*HEC2*	*3.00E-39*	*bHLH*	*NI*
MEDTR6G044600.1	PHD-finger family protein	AT1G43770.2	RING/FYVE/PHD zinc finger protein	1.00E-30	PHD	*NI*
AC233577_2.1	Common plant regulatory factor 1	AT2G46270.1	GBF3	2.00E-52	bZIP	*NI*
*MEDTR2G086420.1*	*TGACG-sequence-specific DNA-binding protein*	*AT1G08320.3*	*TGA9,bZIP21*	*1.00E-169*	*bZIP*	*NI*
MEDTR7G104190.1	Transcription factor HBP-1a	AT1G19490.1	bZIP transcription factor family protein	1.00E-44	bZIP	*NI*
**MEDTR7G104480.1**	***ABSCISIC ACID-INSENSITIVE 5***	**AT2G36270.1**	**ABI5,GIA1**	**1.00E-93**	**bZIP**	***NI***
AC231371_8.1	Transcription factor TGA5	AT4G18650.1	transcription factor-related	7.00E-58	bZIP	ABI3
MT3.5.1S042358	Common plant regulatory factor 1	AT2G46270.1	GBF3	1.00E-31	bZIP	*NI*
MT3.5.1S030865	Probable salt tolerance-like protein At1g78600	AT1G78600.1	LZF1,STH3,DBB3	5.00E-76	C2C2(Zn)Co	ABI3/ABI5
MEDTR4G071590.1	GATA transcription factor 7	AT4G36240.1	GATA7	3.00E-45	C2C2(Zn)GATA	*NI*
MEDTR7G112330.1	GATA transcription factor 16	AT5G49300.1	GATA16	3.00E-24	C2C2(Zn)GATA	ABI3
MEDTR5G061270.1	Zinc finger protein 622	AT4G31420.2	Zinc finger protein 622	2.00E-55	C2H2	ABI3
MEDTR5G088760.1	Dr1-associated corepressor	AT3G12480.1	NF-YC11	7.00E-41	CCAAT	*NI*
*MT3.5.1S062819*	*Nuclear transcription factor Y subunit B-3*	*AT4G14540.1*	*NF-YB3*	*2.00E-46*	*CCAAT*	*ABI3*
MT3.5.1S010268	Zinc finger CCCH domain-containing protein 2	AT1G03790.1	SOM	2.00E-71	CH3	ABI3
MEDTR4G078660.1	Zinc finger CCCH domain-containing protein 2	AT1G03790.1	SOM	6.00E-64	CH3	*NI*
MEDTR3G087530.1	ETHYLENE INSENSITIVE 3-like 5 protein	AT5G65100.1	Ethylene insensitive 3 family protein, EIN3-like 5	1.00E-100	*EIL3*	ABI3/ABI5
MEDTR2G082090.1	Chitin-inducible gibberellin-responsive protein 1	AT1G50600.1	SCL5	1.00E-144	GRAS	*NI*
MEDTR7G086940.1	WUSCHEL-related homeobox 11	AT3G03660.1	WOX11	1.00E-52	HB	ABI5
*MT3.5.1S020015*	*Homeobox-leucine zipper protein HAT3*	*AT3G60390.1*	*HAT3*	*3.00E-83*	*HD-ZIP*	*NI*
**MT3.5.1S062160**	**Heat shock factor protein HSF30**	**AT2G26150.1**	**ATHSFA2,HSFA2**	**9.00E-63**	**HSF**	**ABI3**
AC232874_1024.1	MADS-box transcription factor 15	AT1G22130.1	AGL104	5.00E-43	MIKC	ABI3/ABI5
*MEDTR5G032520.1*	*MADS-box protein SVP*	*AT2G22540.1*	*SVP,AGL22*	*9.00E-89*	*MIKC*	*ABI3*
*MT3.5.1S064922*	*Myb-related protein Myb4*	*AT3G61250.1*	*AtMYB17,MYB17, LMI2*	*1.00E-72*	*MYB*	*ABI3*
**MEDTR3G088110.1**	**NAC domain-containing protein 2**	**AT1G01720.1**	**ATAF1,ANAC002**	**1.00E-116**	**NAC**	**ABI3**
*MEDTR3G116070.1*	*Protein CUP-SHAPED COTYLEDON 3*	*AT1G76420.1*	*CUC3,NAC368,ANAC031*	*1.00E-75*	*NAC*	*ABI5*
**MT3.5.1S030181**	**NAC domain-containing protein 72**	**AT4G27410.2**	**RD26,ANAC072**	**4.00E-99**	**NAC**	**ABI3**
MEDTR5G015680.1	Speckle-type POZ protein-like B	AT5G67480.1	FSH/Ring3 class transcription regulators with BTB/POZ and TAZ domains	1.00E-141	BTB, TAZ POZ domain	*NI*
*MEDTR3G106030.1*	*Transcription factor*	*AT4G31805.1*	*POLAR*	*4.00E-14*	*WRKY*	*NI*
***MEDTR8G020560.1***	***GRL2 (fragment)***	***AT3G13960.1***	***AtGRF5***	***7.00E-53***	***GRF***	***NI***
*MT3.5.1S051069*	*Global transcription factor group E6 homolog*	*AT3G52280.1*	*GTE6*	*6.00E-93*	*Bromo-domain containing*	

*Characters in bold indicate transcription factors involved in drought and/or ABA signaling. Those in italics indicate transcription factors involved flowering, cell transition and cell fate/identity. Transcripts with deregulated expression by MtABI3 and/or MtABI5 are indicated. NI, not identified*.

Strikingly, 17 TF (35%) with increased transcript levels in relation to DT were homologs of *Arabidopsis* regulators that are broadly associated with cell fate or cell identity (see italics characters in Table 1). For example, TRANSCRIPTION FACTOR GROUP E6 (GTE6) regulates differences in leaf patterning between juvenile and mature leaves. PLETHORA 2 (PLT2) is a master regulator of embryonic pattern formation during embryogenesis and root development during seedling growth (Galinha et al., 2007). CUP-SHAPED COTYLEDON 3 (CUC3) regulates embryonic shoot meristem formation and cotyledon boundary specification (Hibara et al., 2006). HAT3 controls apical embryo development and meristem function (Turchi et al., 2013). GRF5 regulates the promotion and/or maintenance of cell proliferation activity in leaf primordia. In rice, the homolog of WOX11 regulates the activation of crown root emergence and growth (Zhao et al., 2009). Yet, during the acquisition of DT during maturation and during PEG, the cell cycle activities are repressed (Faria et al., 2005). Whether these TF participate at the repression of these activities or whether they are necessary to coordinate the resumption of growth and development during rehydration remains to be assessed.

The *Medicago* TF list also contains homologs of transcriptional activators of *Arabidopsis* that participate in the precise regulation of the timing of flowering by incorporating temperature or day length information to promote floral transition and flower meristem identity (Table 1). Among these, we find bHLH80 and bHLH130, respectively, homologs of FLOWERING BHLH 1 and 4 (FBH1 and FBH4), MADS-box protein SVP (homolog of SHORT VEGETATIVE PHASE SVP) and Myb-related protein MYB4 (homolog of MYB17) (Gregis et al., 2008; Jang et al., 2009; Zhang et al., 2009; Ito et al., 2012). The implication of regulators integrating light information or controlling flowering was further reinforced by the identification of homologs of CHO1 (CHOTTO1) and SOM (SOMNUS) (Kim et al., 2008; Yano et al., 2009) and, in the DT-UP list, the photoreceptor FLAVIN-BINDING KELCH REPEAT F BOX 1(FKF1). Although our analysis is based solely on the comparison of transcript levels, and care should be taken with the interpretation of the data based on *Arabidopsis* homologs, altogether, these findings raise new questions about the mechanisms involved in the regulation of DT and developmental transitions. One such question is whether these regulators could play a role in integrating developmental and environmental signals such as light to maintain irreversible the transition from a desiccation-sensitive to a tolerant stage.

If DT has integrated regulatory pathways that relay developmental and environmental cues, then this should be revealed in the *cis*-regulatory elements of promoter genes of the DT-UP list. For 393 out of 740 transcripts of the DT-UP list, promoters could be retrieved using the IMGAG Mt3.5.1 version. The most abundantly represented *cis*-elements were related to ARBRE elements (Table 2): 213 out of 393 promoters contained ABRE-related sequences (ABRERATCAL). These elements are targets of the bZIP ABI5 or ABRE binding factors, which suggests a role of these TF in DT in *M. truncatula* seeds. Many promoters of the DT-UP genes also contain DRE elements, but they were not significantly over-represented. DRE regulatory *cis*-elements known to regulate ABA-dependent and -independent gene expression in response to osmotic stress and their role in seeds has been suggested (Fujita et al., 2011). Numerous genes contained *cis*-elements that are related to light mediated regulation/“G box” (GBOXLERBCS), known for binding with GBF, “BoxII” (LREBOXIIPCCHS1), “LRE” (LRENPCABE), and “Sequences Over-Represented in Light-Induced Promoters” (SORLIP4AT). The c*is*-element GBOXLERBCS was also found enriched in the DT transcriptome of PEG-treated germinated seeds of Arabidopis (Maia et al., 2011), and supports our hypothesis that regulation of DT has adopted regulatory pathways integrating light signals. Two overrepresented *cis*-regulatory elements were related to auxin, AUXREPSIAA4 and SGBFGMGMAUX28. This is consistent with the observation that several genes involved in cell identity present in the DT-list (PLT2, GRF5, Table 1) mediate auxin responses. Twenty seven genes contained the Sucrose Responsive Element 2 (SURE2), a motif conserved among genes regulated by sucrose (Grierson et al., 1994). Finally, 27 genes contained a UPRMOTIFIIAT “Motif II,” a conserved UPR (unfolded protein response) cis-acting element in *Arabidopsis genes* coding for stress proteins (Martinez and Chrispeels, 2003, Table 2).

**Table 2:** **Indentification of *Cis*-acting elements in promoters of genes correlated with desiccation tolerance in *Medicago truncatula***.

**Binding site ID**	**Sequence pattern**	**DT-UP list**		**Random dataset**	
		**# of elem.**	**% of genes**	**# of elem.**	**% of genes**	***P*-value**
ABADESI1	RTACGTGGCR	7	1.8	4	0.3	6.26E-04
ABREATCONSENSUS	YACGTGGC	50	13.1	70	5.0	2.29E-08
ABREATRD22	RYACGTGGYR	39	10.2	35	2.5	3.12E-08
ABRERATCAL	MACGYGB	213	55.6	571	40.7	1.86E-07
ACGTABREMOTIFA2OSEM	ACGTGKC	142	37.1	206	14.7	1.06E-22
GADOWNAT	ACGTGTC	95	24.8	114	8.1	5.32E-20
ACGTOSGLUB1	GTACGTG	35	9.1	66	4.7	2.26E-19
GBOXLERBCS	MCACGTGGC	27	7.0	48	3.4	1.70E-03
BOXIIPCCHS	TCCACGTGGC	77	20.1	120	8.6	1.60E-10
LRENPCABE	ACGTGGCA	51	13.3	79	5.6	2.88E-07
SORLIP4AT	GTATGATGG	5	1.3	13	0.9	5.11E-01
AUXREPSIAA4	KGTCCCAT	28	7.3	56	4.0	6.54E-03
SGBFGMGMAUX28	TCCACGTGTC	16	4.2	4	0.3	1.40E-10
SURE2STPAT21	AATACTAAT	33	8.6	58	4.1	2.64E-04
CACGTGMOTIF	CACGTG	127	33.2	235	16.7	1.44E-12
EMBP1TAEM	CACGTGGC	36	9.4	54	3.8	1.08E-05
IRO2OS	CACGTGG	83	21.7	111	7.9	1.72E-14
QARBNEXTA	AACGTGT	71	18.5	176	12.5	2.60E-03
UPRMOTIFIIAT	CC(N)12CCACG	27	7.0	48	3.4	1.70E-03

*Promoter enrichment analysis of 382 up-regulated genes from the desiccome (DT-UP list, Figure 1) using 1.5Kb promoter regions upstream of the translational start. DNA motifs were counted using PLACE database resource (http://www.dna.affrc.go.jp/PLACE/signalup.html). #, mumber of cis-elements in the indicated data sets*.

### A new player in desiccation tolerance: the bZIP transcription factor *MtABI5*

**Figure 3:**
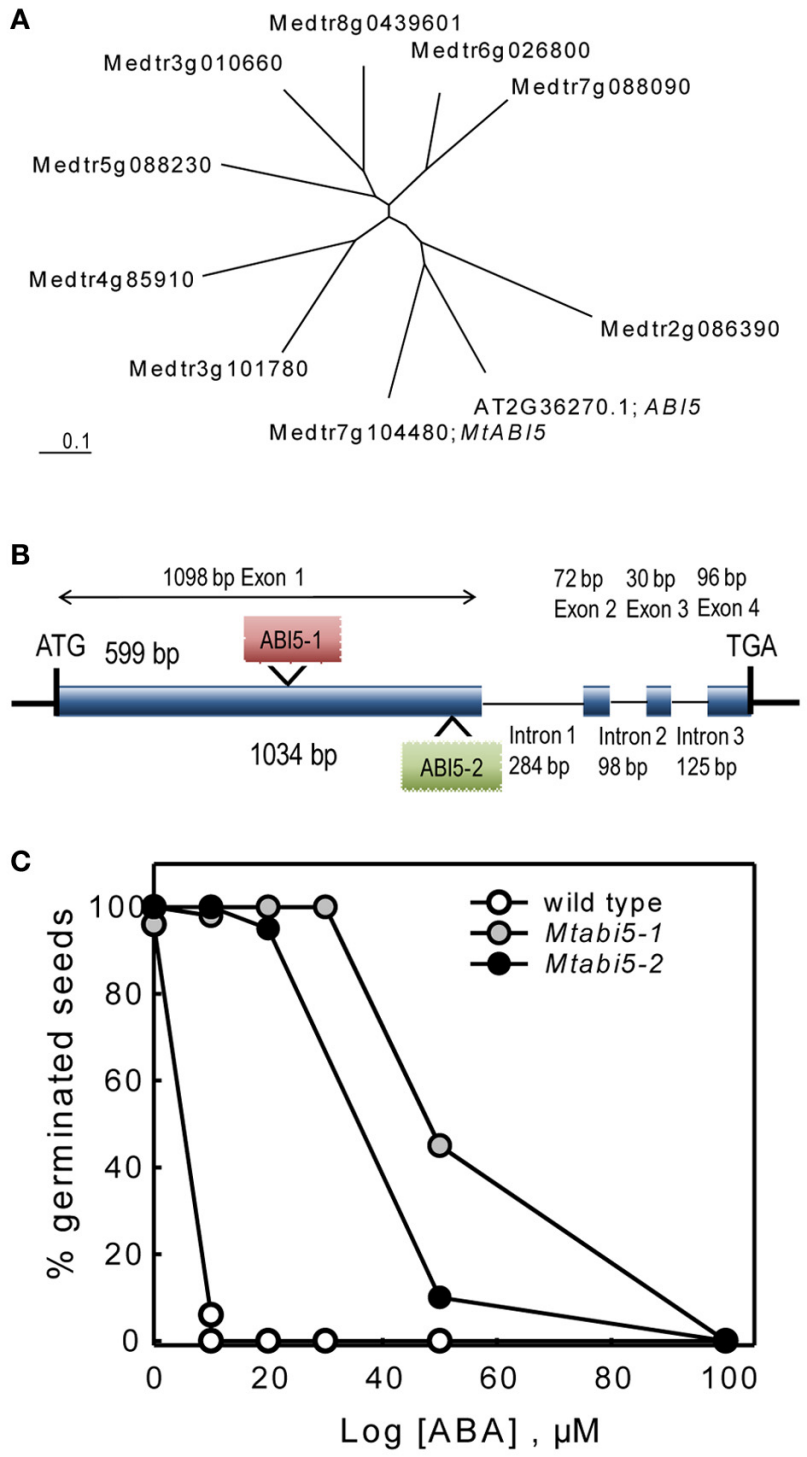
**Characterization of *abscisic acid insensitive* 5 (*Mtabi5*) mutants of *Medicago truncatula*. (A)** Unrooted tree showing *MtABI5* and closest homologs together with the A*rabidopsis thaliana* ABI5. CLUSTALW was used for alignment and TREEVIEW for graphical output. Gene identifiers correspond to IMGAG Mt3.51 **(B)** Gene structure of *MtABI5* with Tnt1 insertions in *Mtabi5-1* and *Mtabi5-2* alleles. **(C)** Percentages of germination of wild type and *Mtabi5* mutant seeds in the presence of ABA. Seeds were scarified before imbibition. Germination (30–50 seeds) was scored as emergence of the radicle after 14 days. Data are the average of duplicates from two independent experiments.

One of the TF that was present in the DT-UP list is a bZIP that is highly homologs to the *Arabidopsis ABI5 gene* (Table 1). In *Arabidopsis*, ABI5 binds to ABRE elements of Em genes whose homologs were found in our DT list (Finkelstein et al., 2005; Cutler et al., 2010; Supplemental Table S1). In concert with ABI3, ABI5 is known to regulate post-germinative growth arrest leading to drought tolerance in *Arabidopsis* seedlings (Lopez-Molina et al., 2001, 2002). This prompted us to test whether *MtABI5* was a key factor in regulating DT and if so, what its target genes are in the desiccome gene set. Considering that the *Arabidopsis genome* comprises nine genes forming a *ABI5*-homologs subfamily of bZIP TF binding to ABRE elements (Fujita et al., 2011), we first determined whether the *Medicago* gene Medtr7g104480 is homologous to ABI5 or other ABI5-like genes. The *Arabidopsis* ABI5 amino acid sequence was blasted against the Mt3.5.1 *Medicago* database and nine sequences were retrieved. A phylogenetic tree was constructed including the *Arabidopsis* ABI5 sequence (Figure 3A) and confirmed that Medtr7g104480 is indeed the closest homolog to ABI5.

The function of *MtABI5* in DT was assessed using two independent homozygous *Tnt1* insertion mutants (*Mtabi5-1* and *Mtabi5-2*, Figure 3B) that were obtained from the insertion collection curated by the Samuel Noble Foundation. The *Tnt1* insertions were located at 599 and 1034 bp from the start-codon, respectively, before the bZIP domain (Figure 3B). After the production of freshly harvested seeds, the ABA sensitivity of both *Mtabi5* mutants was tested (Figure 3C). Like for *abi5* mutants of *Arabidopsis* (Lopez-Molina et al., 2001), mature *Mtabi5* seeds exhibited a strongly reduced sensitivity to ABA. In the presence of 10μM, *Mtabi5* seeds germinated at 100% whereas germination was fully inhibited in wild type seeds at this concentration.

**Figure 4:**
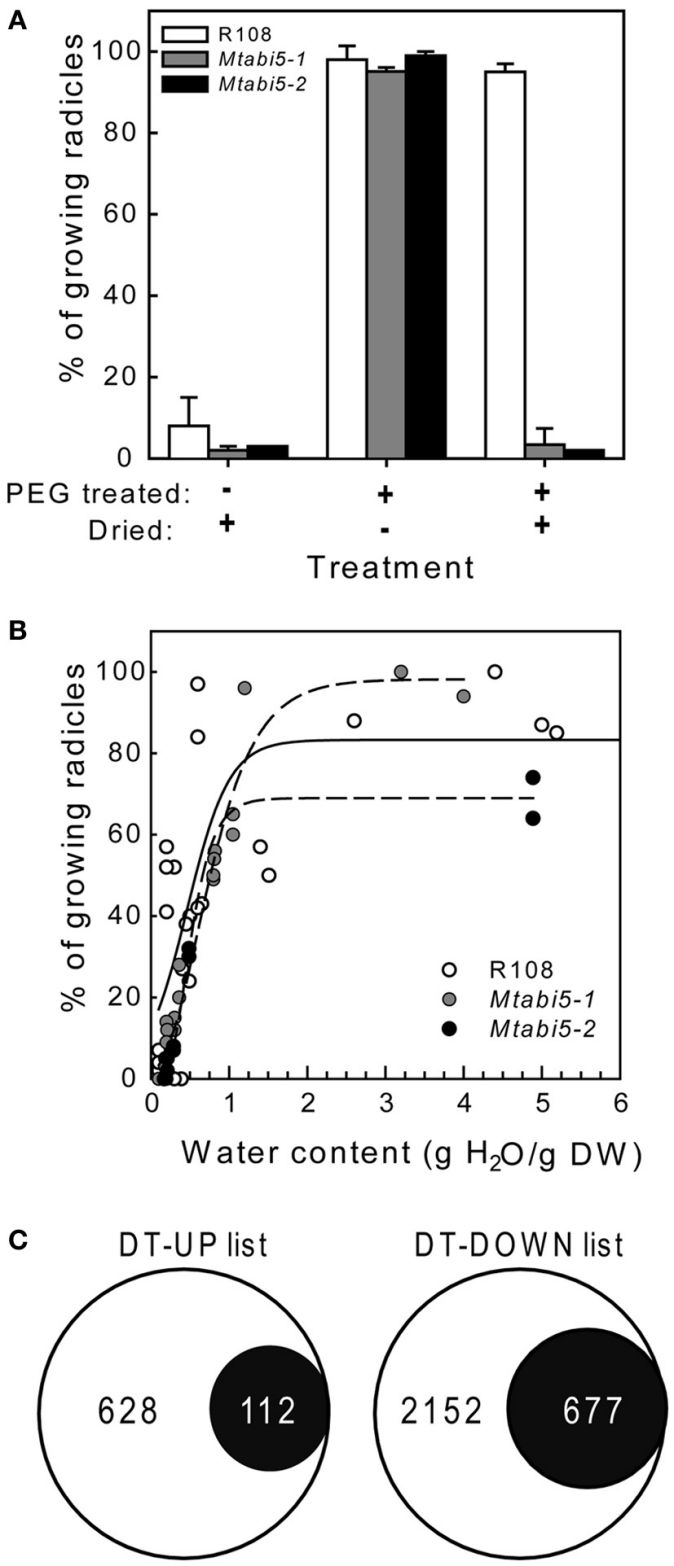
**Desiccation tolerance is not re-established during PEG treatment in germinated *Mtabi5* seeds. (A)** Percentages of desiccation tolerance of 2.7 mm long protruded radicles from germinated wild-type and *Mtabi5* seeds. Seeds were dried at 42% relative humidity (RH) for 3 days before or after a PEG treatment consisting in incubating seeds for 3 days in a polyethylene glycol (PEG) solution (−1.7 MPa) at 10°C. Data represent the average (±SE) of three independent experiments of 50 seeds. **(B)** The effect of drying at 42% RH at the indicated water content on survival of 2.7 mm long protruded radicles of germinated seeds. Each data points represent 30 seeds from three independent experiments. **(C)** Euler diagrams showing the number of transcripts in the DT-UP and DT-DOWN list (Figure 1) that do not change significantly in germinated *Mtabi5* radicles after PEG incubation.

Scarified, dry mature seeds of the *Mtabi5* mutants were all able to germinate when imbibed at 20°C on wet filter paper, indicating that they acquired DT. Next, we tested whether ABI5 was implicated in the re-establishment of DT in sensitive emerged radicles during an osmotic treatment at −1.7 MPa using a PEG solution. In germinated wild type seeds, DT of emerged radicles is lost progressively during growth from 1 to 3 mm (Buitink et al., 2003). Control experiments confirmed that germinated seeds of the *Mtabi5* mutants were not desiccation tolerant (Figure 4A). Furthermore, like germinated untreated wild-type seeds, the germinated *Mtabi5* mutants started to lose their viability when water content decreased below 1.0 g H_2_O g DW^−1^ and decreased sharply below 0.5 g H_2_O g DW^−1^ (Figure 4B). The comparable critical water content between germinated *Mtabi5* and wild type seeds suggests that *Mtabi5* seeds are not more sensitive to drought. Next, we tested whether germinated *Mtabi5* seeds exhibiting a 2.7 mm long emerged radicle were able to tolerate the osmotic stress (−1.7 MPa) brought by the PEG treatment that is necessary to re-establish DT in wild type seeds. Figure 4A shows that after 3 days in PEG, germinated *Mtabi5* seeds were able to resume growth when transferred into water. However, when germinated, PEG-treated *Mtabi5* seeds were dried to water contents of 9% (dry weight basis), they were no longer able to resume growth upon rehydration, indicating that the *Mtabi5* seeds remain desiccation sensitive, and lost their competence to re-establish DT upon osmotic treatment (Figure 4A).

Our data show that *Mtabi5* seeds acquire their DT during maturation but were unable to re-establish it after germination. Although the temporal acquisition of DT might differ between *Mtabi5* seeds and wild type seeds during seed development, our data suggest that *MtABI5* is much more a critical determinant for DT in seedlings than during maturation. Possibly, there might be redundant pathways in place during maturation that are absent in germinated seedlings. Indeed ABI5 and other bZIP factors of the same class operate with many other TF in a complex combinatorial control of gene expression that is poorly understood (Nakamura et al., 2001; Lopez-Molina et al., 2002; Finkelstein et al., 2005; Cutler et al., 2010; Lindemose et al., 2013; Verdier et al., 2013).

To characterize the part of the desiccome that is influenced by *MtABI5* during the re-establishment of DT tolerance, a transcriptome analysis was performed on the protruded radicles of germinated *Mtabi5* mutants before and after the incubation in the PEG solution (Figure 4C). A total of 852 out of the 3626 transcripts with increased levels in the wild type were no longer up-regulated by the PEG treatment in the *Mtabi5* mutant (*P* > 0.01 and ratio after PEG/before PEG < 1 or > −1). Likewise, out of the 6691 transcript levels that decreased during the PEG treatment in the wild type, 1865 no longer decreased in the *Mtabi5* mutants. Next, these data were compared to the DT-UP and DT-DOWN lists (Figure 4C, Supplementary Table S2). 112 of the 740 transcripts (15%) that are present in the DT-UP list were no longer up-regulated in the *Mtabi5* mutants whereas 23% (677) of the 2829 transcripts of DT-DOWN list were no longer down-regulated. From these data, *MtABI5* seems to play a predominant role as a repressor rather than an activator during the re-establishment of DT. Further work is needed to investigate which of these genes are under the direct regulation of *MtABI5* or modulated downstream of *MtABI5* pathways.

### Transcriptome analysis identifies *MtABI3*-related genes involved in DT

**Figure 5:**
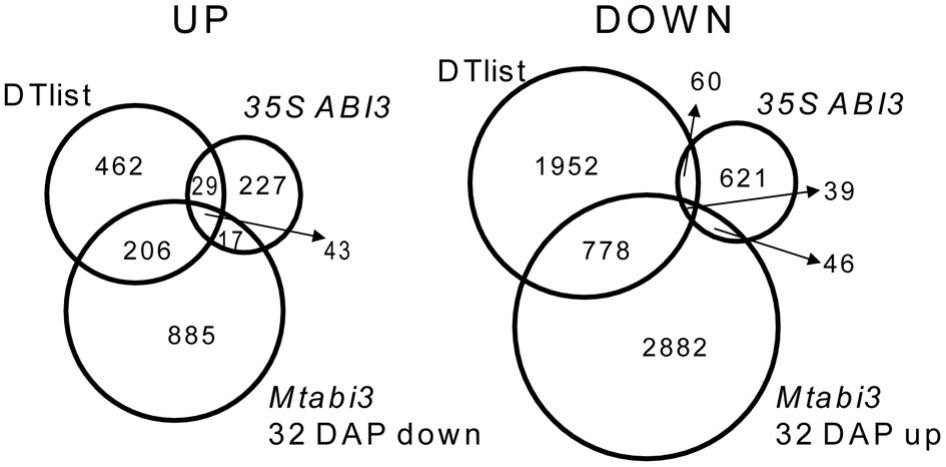
**Venn diagram identifying DT transcripts related to *ABSCISIC ACID 3 (ABI3*) of *Medicago truncatula*.** Transcripts that were identified in the DT-UP or DT-DOWN lists were compared to those that are differentially expressed in *Mtabi3* seeds at 32 DAP compared to wild type seeds (*Mtabi3* 32 DAP) and to those that are differentially expressed upon ectopic expression of *MtABI3* in hairy roots compared to control. Transcript levels were considered significant when *P* < 0.01 and −1 > Mlog2 > 1.

A recent characterization of Tnt1 *abi3* mutants of *Medicago* demonstrated that like for *Arabidopsis*, *Medicago abi3* mutant seeds are desiccation sensitive and contain reduced amount of LEA proteins (Delahaie et al., 2013). To investigate which genes of the desiccome were regulated directly or acting downstream of *MtABI3*, we took advantage of recently published transcriptome data of developing desiccation-sensitive *Mtabi3* mutant seeds (Verdier et al., 2013, GEO database accession GSE44291). These seeds were analyzed at 32 DAP and compared to wild type seeds of the same age, after DT had been acquired (Verdier et al., 2013). In addition, we compared this dataset to that of the transcriptome of *Medicago* roots ectopically expressing the genomic sequence of *MtABI3* (*35S::ABI3*, Verdier et al., 2013; GEO database accession GSE44291). Amongst the 1151 transcripts with significantly decreased levels in the *Mtabi3* seeds, 249 were present in DT-UP list (i.e., 33% of the list, Figure 5, Supplementary Table S3) and 43 were present in the three data sets. Similarly, out 3745 transcripts that increased significantly in the *Mtabi3* seeds, 817 were present in the DT-DOWN list (i.e., 28%) and 39 were found in common between the three data sets (Figure 5, Supplementary Table S3). To further validate the *MtABI3*-mediated expression of DT genes, we verified whether their *Arabidopsis* homologs were among the list of the experimentally validated targets of the *Arabidopsis* ABI3 regulon (Mönke et al., 2012). For the 43 up-regulated genes present in all three dataset (Figure 5), 19 *Arabidopsis* homologs were identified as part of the regulon of *MtABI3*. Genes in this list encoded LEA proteins and genes involved in seed storage deposition such as oleosins and seed storage proteins. It is noteworthy that transcripts of two cystathionine beta-synthase (CBS) domain proteins were also identified, one of them being *MtSNF4b*, a subunit of the metabolic regulator SUCROSE NONFERMENTING RELATD KINASE (SnRK1). MtSNF4b plays a role in seed longevity and activation of biotic stress pathways in dormant seeds (Rosnoblet et al., 2007; Bolingue et al., 2010). Since Mönke et al. (2012) did not investigate targets that are repressed by ABI3, a comparison was impossible (Figure 5).

### Analysis of ABI3- and ABI5-specific datasets in relation to DT

**Figure 6:**
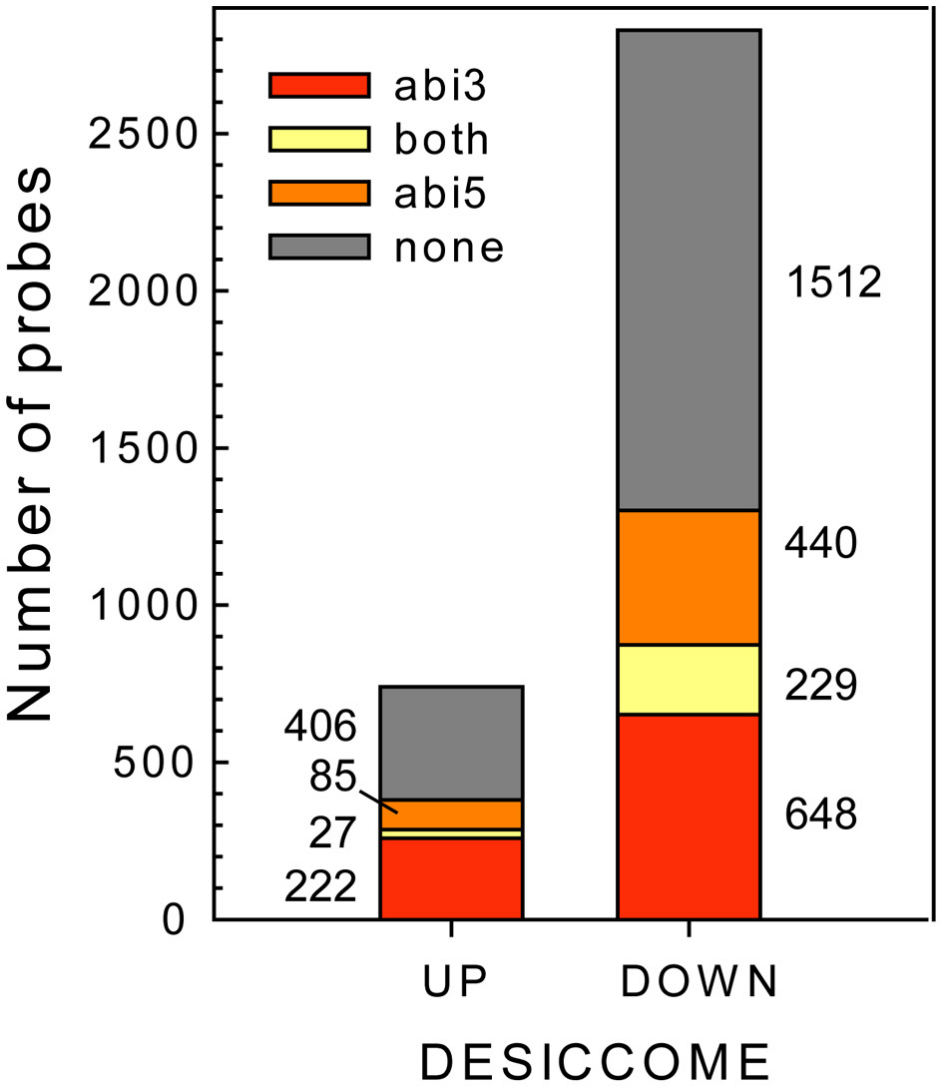
**Identification of ABI3- and/or ABI5 related genes in the desiccome (DT-UP and DT-DOWN lists)**.

Having identified the transcripts that are affected in the *Mtabi3*- and *Mtabi5* mutants in relation to DT, we investigated the putative function of the genes that modulated by one or both TFs (Figure 6). It should be noted that the change in transcript levels is not necessarily through a direct action of ABI3 and or ABI5, since transcript levels can be modified by downstream targets of these TFs or through other mechanisms than transcriptional activation. However, to facilitate the reading, we refer hereafter to ABI3 or ABI5-related genes. The putative regulation of transcripts by these two TFs is annotated in Table 1 and Supplemental Table 1. A GO-enrichment analysis was performed on these gene lists (Table 3).

**Table 3:**
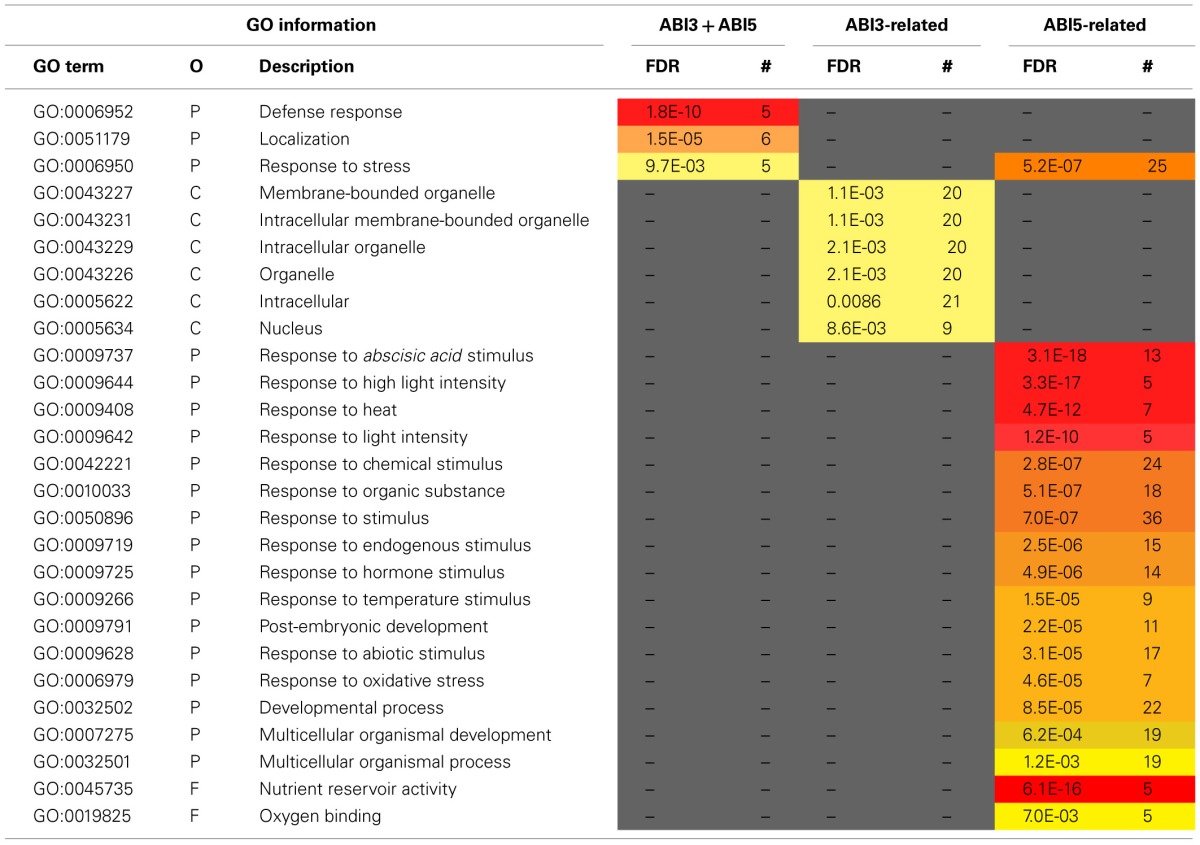
***MtABI3* and *MtABI5* are involved in complementary processes in relation to desiccation tolerance in *Medicago truncatula***.

*Gene Ontology (GO) enrichment analysis on the up-regulated genes related to desiccation tolerance (DT-UP list, Figure 1) that are deregulated by MtABI5 and/or MtABI3. P-values of the false discovery rate (FDR) are shown together with the number of genes (#). Analysis was performed using AgriGO with the Arabidopsis TAIR9 background applying a Chi^2^ statistical test with the Yekutieli multi-test adjustment method. P, Biological process; C, component; F, molecular function*.

Only a small amount of transcripts are influenced by both TFs, representing 4 and 7% of the DT-UP and DT-DOWN lists, respectively (Figure 6). Among the 27 genes from the DT-UP list that are downstream of both TFs, we noticed three different glutamate-cysteine ligases that catalyze the first and rate limiting step in the biosynthesis of glutathione (GSH, Noctor et al., 2012) (Supplementary Table S1). In *Arabidopsis*, *gsh1* mutants confer a recessive embryo-lethal phenotype and it was suggested that the autonomous synthesis of GSH in the embryo was necessary for proper seed maturation (Cairns et al., 2006). During drying, the resurrection plant *Sporobolus stapfianus* exhibited an important increase in GSH and γ-glutamyl amino acids compared to the desiccation-sensitive *Sporobulus pyramidalis* (Oliver et al., 2011). Thus, GSH might represent a key antioxidant involved for the survival in the dry state. A RT-qPCR study validated their expression level (Supplemental Table S4). Surprisingly, LEA transcripts were found to be regulated only by ABI3; in the *Mtabi5* mutants they increased in a comparable way to the wild type radicles (Supplementary Table S3). Four TFs were related to both *MtABI3* and *MtABI5* (Table 1). It is noteworthy that homologs of two of them mediate light responses in *Arabidopsis*; FBH1 and a gene bearing high homology to LZF1 (LIGHT-REGULATED ZINC FINGER PROTEIN 1). In *Arabidopsis*, LZF1 is a positive regulator functioning in de-etiolation and the accumulation of anthocyanin (Chang et al., 2008).

The pathway downstream of ABI3 is more affected compared to ABI5, representing, respectively, 30% (222 transcripts) of the DT-UP list and 23% (648 transcripts) of DT-DOWN list. Biological processes that were overrepresented in the ABI3-related gene list were “response to *abscisic acid* stimulus,” including ABA responsive genes such as homologs of RD26 or PROTEIN-L-ISOASPARTATE METHYLTRANSFERASE 1 (PIMT1), playing a role in protein repair after seed ageing (Ogé et al., 2008) (Table 3). The GO term “response to heat” included heat stress associated proteins, HSP (HSP101, sHPs) and HSF, whereas the GO term “multicellular organismal development” contained ABI3 targets, such as storage reserve proteins, 1-cys-peroxiredoxin and LEA genes.

Out of the 85 transcripts corresponding to *Medicago* genes that were identified as up-regulated via *MtABI5*, only few were annotated (Supplemental Tables S1, S2). Many of them corresponded to sequences implicated in transcriptional regulation (DNA polymerases, RNA processing) and post-transcriptional regulation (ubiquitin-related). Several bHLH TFs were identified, namely bHLH135 (PRE5), bHLH84, and bHLH130 (FBH4). The *Arabidopsis* FBH4 positively regulates CONSTANS transcription for photoperiodic flowering together with FBH1 (Ito et al., 2012).

A GO enrichment analysis on the DT-DOWN list revealed that the main biological processes for which transcripts are down-regulated by both TFs are cell cycle, secondary metabolic processes and specific kinase or receptor signaling pathways (Table 4). The ABI5-related dataset showed a highly significant overrepresentation of processes related to cell division and actin cytoskeleton organization. This category goes together with the previously mentioned *CUC3* TF whose expression is affected by ABI5 and involved in the repression of cell division (Table 1). Another biological processes enriched in the ABI5-related probe list is “defense response,” including genes involved in flavonoid and phenylpropanoid biosynthesis and programmed cell death. The GO analysis of the overrepresented biological processes of the down-regulated transcripts that are related to ABI3 is complementary to that of the ABI5-related list (Table 4). ABI3 appears to regulate biological processes involved in “DNA replication,” “cell wall modification” and genes involved in microtubule-based processes. Interestingly, this goes together with the ABI3-dependent up-regulation of PHS1 (PROPYZAMIDE-HYPERSENSITIVE 1), involved in phosphorylation cascades that control the dynamics of cortical microtubules in plant cells (Naoi and Hashimoto, 2004). *Phs1* mutants are hypersensitive to ABA. In addition, other biological processes were related to primary metabolic process, such as fatty acid biosynthetic process, carbohydrate metabolic process, and lipid biosynthetic process. A final category is related to DNA repair.

**Table 4:**
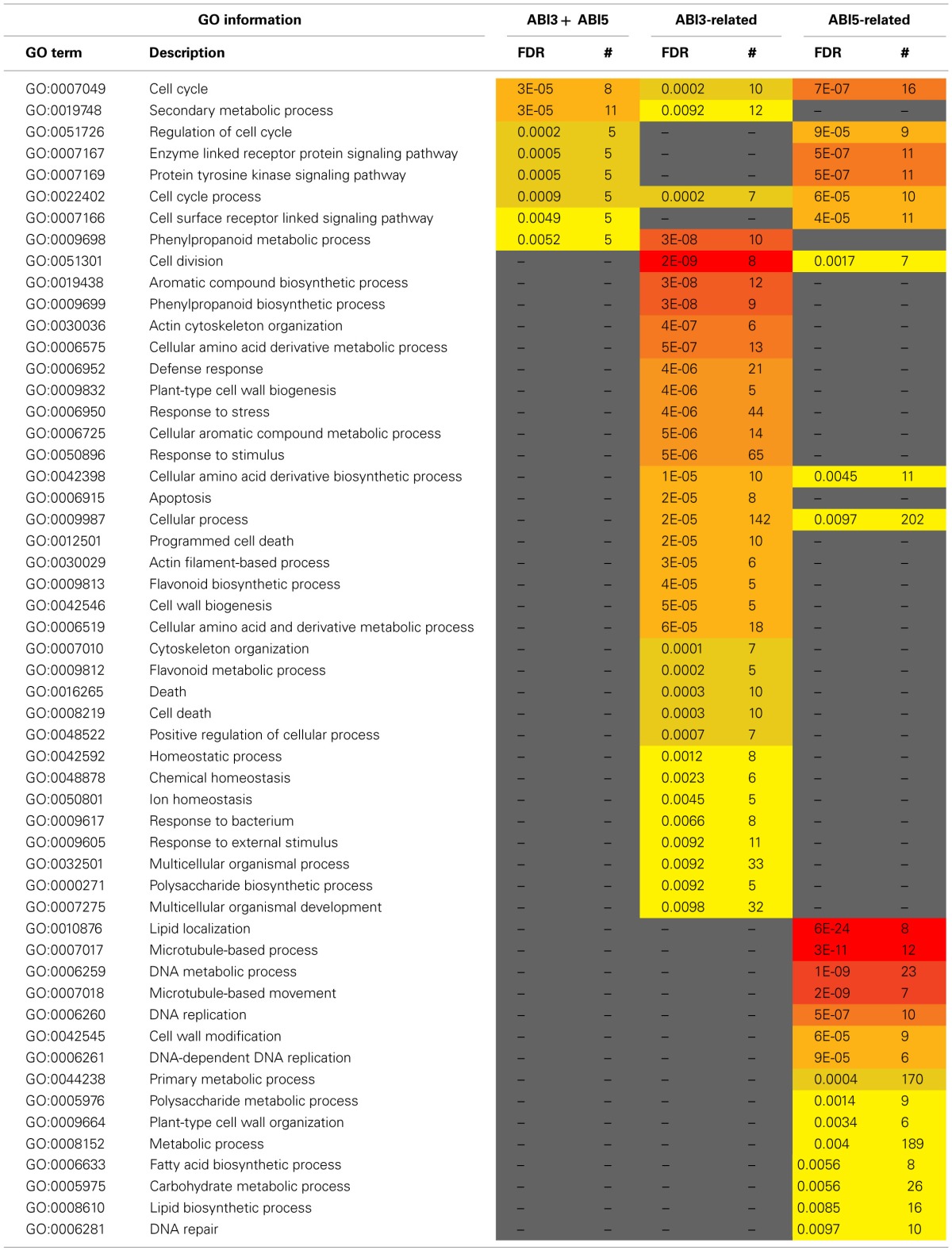
***MtABI3* and *MtABI5* are involved in complementary processes in relation to desiccation tolerance in *Medicago truncatula***.

*Gene Ontology (GO) enrichment analysis on the down-regulated genes related to desiccation tolerance (DT-DOWN list, Figure 1) that are deregulated by MtABI5 and/or MtABI3. P-values of the false discovery rate (FDR) are shown together with the number of genes (#). Analysis was performed using AgriGO with the Arabidopsis TAIR9 background applying a Chi^2^ statistical test with the Yekutieli multi-test adjustment method. P, Biological process; C, component; F, molecular function*.

## Concluding remarks

The transcriptome comparisons combining two developmental contexts during which DT is acquired, together with the transcriptome analysis of two desiccation sensitive mutants generated many intriguing hypotheses related to the regulatory mechanisms that determine DT. We are aware that the hypotheses described in this study are based solely on steady-state transcript levels and inferred from homologous genes characterized in *Arabidopsis*. Therefore, more work is necessary to define gene functions and dissect the complex regulation of gene expression. In addition, post-transcriptional and post-translational processes need to be taken into account. Yet, the data suggest that the regulatory pathways leading to DT act redundantly and respond to both developmental and environmental cues. None of the monogenic mutation in the 49 TF gene that were identified in the desiccome induced lethal or desiccation sensitive phenotypes in *Arabidopsis*, except for ABI3. These TFs are likely acting redundantly depending of the environmental context. Our data suggest that ABI3 and ABI5 have complementary roles in DT and act in a complex combinatorial control of gene expression (Nakamura et al., 2001; Finkelstein et al., 2005; Cutler et al., 2010; Lindemose et al., 2013).

The desiccome contained a surprisingly high number of transcripts for which *Arabidopsis* homologs are involved in the control of flowering, cellular phase transitions and cell identity. These data suggest that DT evolved by coopting existing genetic pathways regulating developmental phase transition and light-sensing. There is increasing evidence in the literature suggesting an interaction between photoperiod and drought stress, whereby photoperiodically- or light-induced genes coordinate the ABA-mediated activation of genes (Chen et al., 2008; Riboni et al., 2013). Therefore, it is important to investigate whether genes regulated by photoperiod or light have a conserved function regulating DT both in seeds as well as resurrection plants.

## Authors contributions

Julia Buitink and Olivier Leprince conceived and designed the experiments and wrote the MS. Emmanuel Terrasson and David Lalanne performed the transcriptome analysis. Emmanuel Terrasson and Benoit Ly Vu carried out the physiological experiments. Julia Zinsmeister performed the qPCR experiments. Emmanuel Terrasson, Karima Righetti, and Sandra Pelletier performed the bioinformatic analyses. Emmanuel Terrasson and Karima Righetti critically reviewed the MS.

## Conflict of interest statement

The authors declare that the research was conducted in the absence of any commercial or financial relationships that could be construed as a potential conflict of interest.

## Supplementary materials

Table S1 **List of transcripts that are differentially expressed in relation to desiccation tolerance (DT-UP and DT-DOWN) list (Figure 1)**.

Table S2 **List of desiccation tolerance (DT) correlated transcripts that are affected by the absence of *MtABI5* compared to wild type seeds during the re-induction of DT**.

Table S3 **List of transcripts that are affected by the absence of *MtABI3* compared to wild type seeds during seed maturation at 32 DAP (*Mtabi3* 32 DAP), or that show increased or decreased transcript levels in hairy roots ectopically expressing *MtABI3* compared to roots with an empty plasmid**.

Table S4 **Transcript levels of two glutamyl cysteine ligase genes (Medtr5g010210.1 and Medtr5g010230.1) after the re-induction of desiccation tolerance in radicles of germinated wild type, *Mtabi5-1* and *Mtabi5-2* mutant seeds by a PEG treatment at −1.7 MPa**.
